# Measuring Predictability of Autonomous Network Transitions into Bursting Dynamics

**DOI:** 10.1371/journal.pone.0122225

**Published:** 2015-04-09

**Authors:** Sima Mofakham, Michal Zochowski

**Affiliations:** 1 Biophysics Program, University of Michigan, 930N University, Ann Arbor, Michigan, United States of America; 2 Department of Physics, University of Michigan, 450 Church St, Ann Arbor, Michigan, United States of America; 3 The R.B. Zajonc Institute for Social Studies, Stawki 5/7, 00–183 Warsaw, Poland; Georgia State University, UNITED STATES

## Abstract

Understanding spontaneous transitions between dynamical modes in a network is of significant importance. These transitions may separate pathological and normal functions of the brain. In this paper, we develop a set of measures that, based on spatio-temporal features of network activity, predict autonomous network transitions from asynchronous to synchronous dynamics under various conditions. These metrics quantify spike-timing distributions within a narrow time window as a function of the relative location of the active neurons. We applied these metrics to investigate the properties of these transitions in excitatory-only and excitatory-and-inhibitory networks and elucidate how network topology, noise level, and cellular heterogeneity affect both the reliability and the timeliness of the predictions. The developed measures can be calculated in real time and therefore potentially applied in clinical situations.

## Introduction

The complex dynamics of brain networks underlies information processing as well as various pathologies. Epilepsy [1,2] and/or Parkinson’s disease [3] are the most prominent examples of rapid autonomous transitions of network level spatio-temporal patterning from normal, largely asynchronous behavior into episodes of synchronous pathological activity that constitute underpinnings of the pathology. While, in the case of epilepsy, a significant fraction of seizures can be treated with medications or invasively with surgery, there is still large number of cases in which patients have to deal with a threat of impending seizures. Therefore it becomes imperative to develop tools which, based upon online monitoring of brain dynamics can predict seizure, warn the patient, and/or optimally, take measures (through controlled drug infusion or electrical stimulation) to counteract dynamical changes in the network dynamics near the foci that lead to seizure onset. There is a wealth of research being conducted that is centered on developing metrics and algorithms that would monitor changes in the brain activity (usually EEG signals or intracranial recordings) and predict impending seizures [4–11]. Existing measures have relatively low success rates providing a lot of false positives or false negatives [12–16]. Others analyzed the activity of network and individual neurons around the epileptic onset [17–22].

In this manuscript we take somewhat different approach. We developed a set of measures to study early spatial features of network reorganization upon impending transition into bursting dynamics. Namely, we investigate whether and under what conditions we can identify and later detect early dynamical signs of transitions from synchronous to asynchronous dynamics in highly simplified settings.

In loose terms we assume that the asynchronous mode of activity corresponds to interictal dynamics while synchronous activity corresponds to seizure itself. While this is clearly an oversimplification the goal of this work is to elucidate universal properties of transitions between those two modes of activity.

To make the settings at all relevant to possible clinical applications we the only information we utilize is the relative spatial positions of the neurons and their spiking activity patterns. This could in practice correspond to multiunit information obtained from two or more depth electrodes placed in the brain. We further assume that we have access to this information in the brain region corresponding to localized seizure foci and that transition in this region alone will generate distributed seizure dynamics. We do not tackle the problem of how does synchronous dynamics spread throughout the brain.

We investigate the afore mentioned transitions within ring of excitatory only or excitatory-and-inhibitory integrate-and-fire neuronal networks. This model has been used for more than a century and still is widely used due to its low computational cost, broad range of applications, simplicity along with accuracy [23,24].

Even though that the LIF model is one of the simplest models of neuronal dynamics it can reproduce number of biologically observed spatio-temporal patterns depending on the connectivity, synaptic weights, inhibitory feedback, noise and heterogeneity. In 1991 Abeles, showed that if network wires randomly, tight temporal synchrony in order of milliseconds could be easily attained [25]. However, Hopfield and Herz studied a network of locally connected integrate-and-fire oscillators neurons and they observed mostly asynchronous dynamics unless very late in the simulation (more than a hundred periods in a network) that invalidate any importance of synchrony in coding information comparing with biological short time scales of decision making [26]. This result was partially explained by D. Hansel et al, who investigated dynamics of purely excitatory homogeneous and fully connected networks of LIF and Hodgkin-Huxley (HH) model [27]. They showed that depending on the type of neuronal Phase Response Curve (PRC) excitation for neurons with type 1 characteristic is mostly desynchronizing, however in neurons with type 2 properties (HH), excitation can lead to synchrony. Campbell & Wang (1999) on the other hand showed that network can reach synchrony much faster than the original estimate (within a few periods), they showed that the time needed to reach this synchrony is a logarithmic function of the network size [28]. At the same time it was recently shown that noise statistics itself could dramatically change neural spiking properties [29].

Brunel on the other hand investigated the effect of added inhibition into the excitatory oscillators [30]. He studied the dynamical properties of a network of sparsely randomly interconnected excitatory and inhibitory spiking leaky integrate-and-fire neurons. He showed that the networks could switch between synchronous and asynchronous activity, consisting of the propagating waves of activity, depending on driving frequency and excitatory-inhibition interactions. Along these lines, Tsodyks, et al. showed that excitatory-inhibitory network of LIF neurons that are interconnected with nonlinear synapses can adopt a synchronous activity associated with population bursts intermittent with long periods of asynchronous activity [31]. These types of transitions have been studied recently within the framework or extreme events [32, 33].

Here, we use small-world paradigm to vary network connectivity within the excitatory and inhibitory neurons [34, 35]. It is known that small-world topologies exhibit complex spatio-temporal dynamics including intermittent transitions to bursting activity [36–38]. Specifically, Roxin observed transitions from asynchronous activity to short-term synchronous bursts for such network topologies [39]. Further, Netoff et al. modeled CA1 and CA3 network interactions using three different neuron models (LIF, Hodgkin Huxley, and a Poisson spike-train model) connected into small-world structure. They observed that the burst seen in the CA3 is due to simultaneous and quick reactivation of recently active neurons that happens trough long distance connections. They observed this mechanism in all three different models, suggesting that the fraction of long distance connections are more crucial for network synchrony than the details of each neurons [37].

Here, we use the afore mentioned network properties to study early dynamical features of transitions from asynchronous to synchronous network state. We test performance of the developed metric for various network types and network structures. We varied connectivity patterns under two conditions: when the transitions are driven by cellular heterogeneities in the network (i.e. variation of cellular parameters), and 2) when transitions are driven by noise aimed to simulate uncorrelated input from other brain regions to the foci. Those two conditions are to simulate internally and externally driven transitions towards bursting. In sections 1.1 and 1.2 we describe the observed spatio-temporal patterning in the excitatory only and in the excitatory-and-inhibitory networks. Then in section 2 we introduce metrics used to quantify properties of transitions from asynchronous to bursting regimes.

## Results

### 1. Characterization of network dynamics

We first characterize the simplified neuronal network dynamics and investigate how distinctive network properties such as the connectivity structure, noise and inhibition can shape its dynamics and influence the properties of transitions between different modes of activity patterns. Here we generally differentiate transitions from/into bursting regime to be driven by noise (modeling uncorrelated input from other parts of the network) and, separately those generated internally by the network, caused by distribution of cell intrinsic frequency.

#### 1.1. Excitatory networks with deterministic and noise driven dynamics

First we investigated the dynamics of a network consisting of 200 integrate-and-fire excitatory neurons in 1D ring structure and examine its spatio-temporal patterning as a function of noise, external current and its underlying connectivity pattern. The neurons are set to fire spontaneously as they are driven by constant current or random input. The three stimulation types are intended to simulate cellular changes due to the intrinsic neuronal excitation (constant current), input coming from other brain modalities (random input), or both.

**Fig 1:**
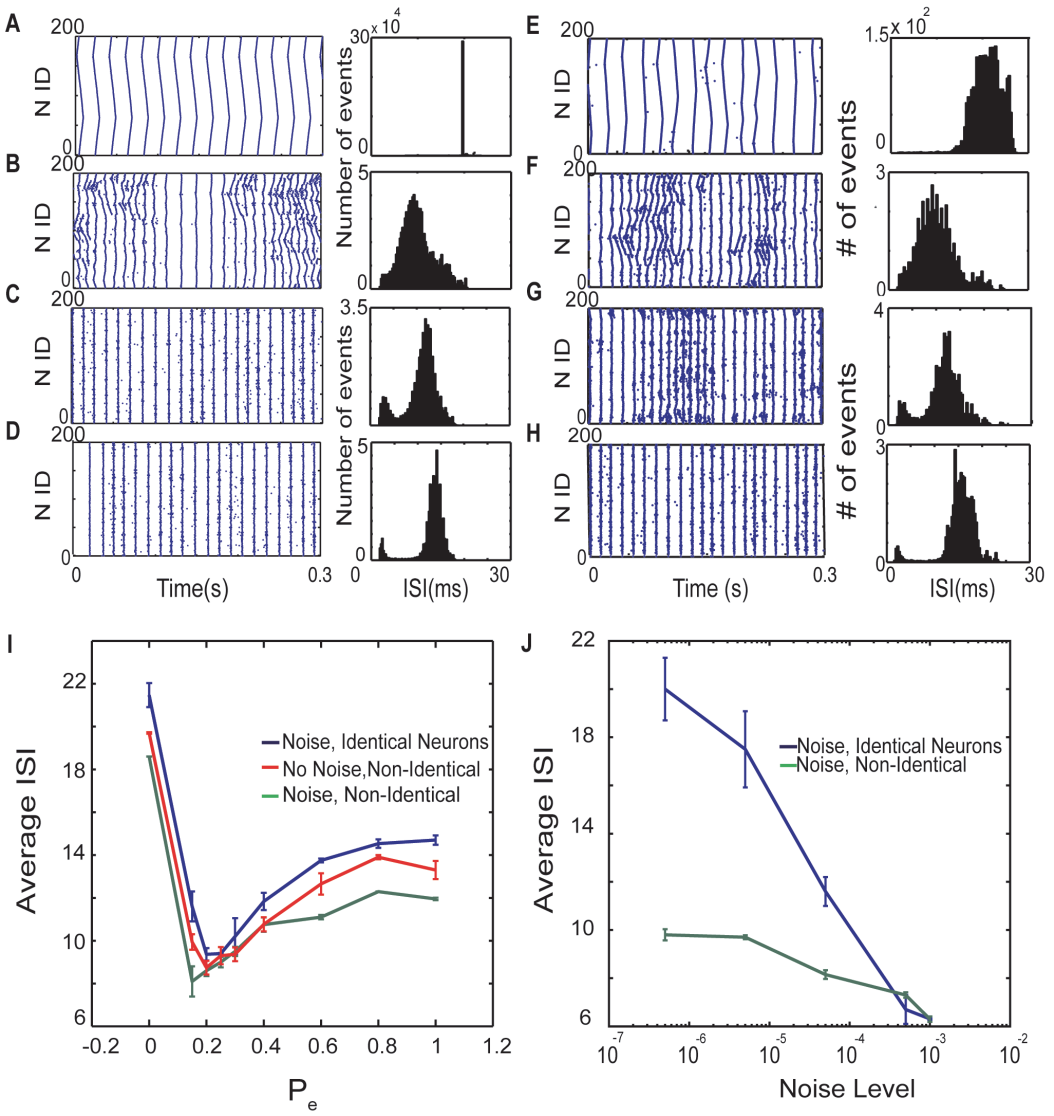
Dynamics of a network of 200 excitatory integrate-and-fire neurons with deterministic (left column) and noise-driven (right column) dynamics. (A-D) Raster plots and ISI histograms associated with deterministic dynamics of networks having P_e_ = 0, 0.15, 0.4 and 1 respectively (blue dots denote timing of neuronal action potential). (E-H) Same as panels (A-D) for noise driven networks (noise frequency = 0.00005). (I) Changes of mean ISIs as a function of rewiring parameter for noise driven identical (I^e^_ext_ = 1.05, f_N_ = 0.00005 blue line), non-identical (I^e^_ext_ = 0.95–1.15, f_N_ = 0.00005, green line) and deterministic dynamics for non-identical neurons (I^e^_ext_ = 0.95–1.15, red line). (J) Changes in mean ISI duration as a function of noise level for an excitatory network with P_e_ = 0.15 for both identical (I^e^_ext_ = 1.05, blue line), and non-identical neurons (I^e^_ext_ = 0.95–1.15, green line). Spike timings used for analysis in this figure is provided as the supplemental data in S1–S8 Dataset.

It is well established that the dynamics of a neuronal network is highly dependent upon its structure; here we use small-world paradigm to vary network connectivity using excitatory rewiring probability P_e._ Accordingly, here we show three major classes of network activity patterns can be formed for local, small-world and random topology in Fig 1A–1D respectively. The panels depict raster plots (left column; blue dots denote action potentials) and histograms (right column) of interspike Intervals (ISIs) associated with fully deterministic dynamics (no noise) of networks having different connectivity patterns. For P_e_ = 0 (Fig 1A), i.e. for networks having exclusively local connectivity, we observe low frequency propagating chains of activity. Given that there is no input noise in the network, and the fact that the activity is initiated by the constant external current, one can observe repetition of the traveling wave-forms over time. The corresponding ISI histogram is very narrow. Fig 1B depicts activity around the small-world regime (P_e_ = 0.15), where most connections are local and few of them are rewired to form long distance connectivity. The Small-world regime is known for high clustering and short path lengths and has been shown that the brain possibly shares these connectivity features [40–45]. The associated dynamics consists of two phases (Fig 1B): 1) short irregular propagating waves of activity that collide occasionally and, 2) globally synchronous activity. At the same time, addition of few random connections causes ISIs to shift toward lower values reflecting the higher firing frequency. These kinds of dynamics were reported earlier and were also observed in various brain modalities during normal function and pathology [39, 46–49]. With P_e_ = 0.4, random connections are frequent enough to transform the dynamics into a single synchronized phase. Interestingly in the ISI histogram we see two distant peaks the main peak corresponds to the dominating low-frequency synchronous activity patterns, whereas the small high-frequency peak is due to the asynchronous activity appearing sporadically. Finally, Fig 1D illustrates networks with exclusively random connections (P_e_ = 1). Where, we observe stable synchronous bursting with frequency much lower than the small-world regime. Fig 1E–1H correspond to the same network structures as those presented in Fig 1A–1D, respectively, but with the addition of the background noise (please refer to methods). Here, the spatio-temporal patterning is similar to the fully deterministic case, with generally shorter episodes of bursting dynamics, more rapid transitions into and out of those regimes and more pronounced episodes of asynchronous dynamics especially for those P_e_ values around the small-world region.

Changes in the mean ISI values as a function of rewiring parameter are plotted for deterministic dynamics of non-identical neurons and noise-driven dynamics of both identical and non-identical cells (Fig 1I). The general pattern for all cases is similar, large ISI for local connectivity pattern (P_e_ = 0.0), followed with a significant drop for small-world connectivity regime (P_e_ = 0.15–0.2), and then increase of ISI values in more random network topologies (P_e_>0.3). This data shows that firing rate is somewhat higher when both heterogeneity and noise present (green line) at P_e_ = 0.15, and it reduced and shifted to P_e_ = 0.2 either with eliminating the noise (red line) or heterogeneity (blue line). Albeit not surprising that the overall frequency increases with addition of noise (additional excitatory input), it is interesting that the frequency changes are more pronounced for random and local network connectivity than the small-world regime. The noise effect on ISIs values for both identical (I^e^_ext_ = 1.05, blue line) and non-identical neurons (I^e^_ext_ = 0.95–1.15, green line) are shown in (Fig 1J). For the intermediate and low values of noise, the cell heterogeneity significantly lowers the average of ISI while for the higher levels of the applied noise there is no significant difference in the firing rates.

**Fig 2:**
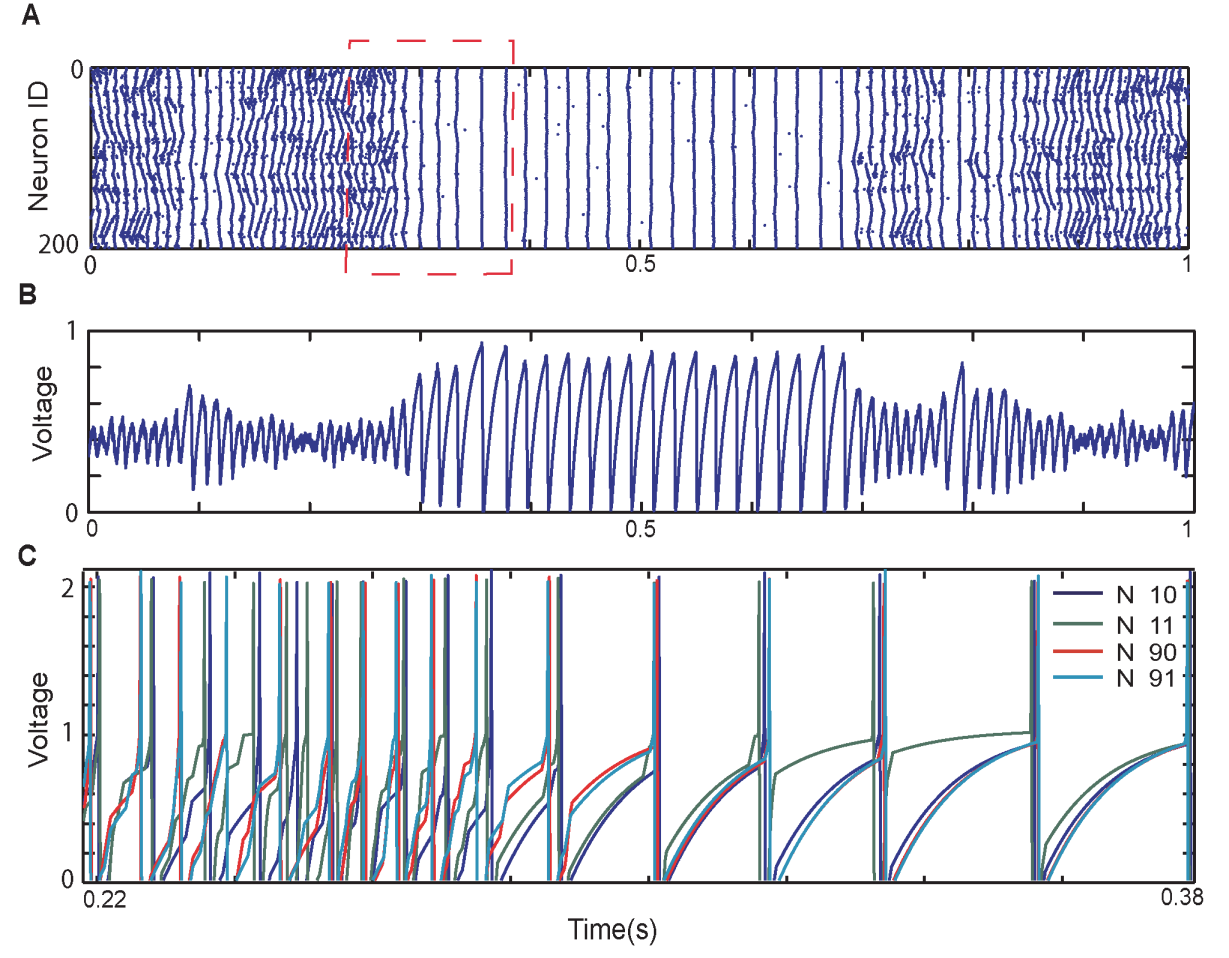
Network activity and individual neurons’ voltage profiles before and after transition into synchronous dynamics. (A, B) An example of raster plot and cumulative network activity pattern for a system composed of 200 excitatory neurons, transitioning from asynchronous to synchronous dynamics. (C) Example of voltage traces of two pairs of neurons ([10, 11],[90,91]) where neurons in each pair are neighbors but the pairs are distant from each other.

Here we will be primarily interested in characterizing transitions between bursting and synchronous activity patterns for different network cellular and network properties. To better illustrate the transitions between the synchronous and asynchronous regime we plotted rasterplot with and example of such transition (Fig 2A) together with cumulative signal of network activity (Fig 2B) and example of voltage traces (Fig 1C) near the transition point. Depicted example corresponds to the network where neurons obtain constant input. We observe, not surprisingly, that the pairs on neurons lying in spatial proximity are generally more synchronous that those positioned far from each other. Furthermore one can observe that during the asynchronous period the dynamics of the pairs is driven by mostly common asynchronous signal causing their activity to desynchronize, while during bursting they respond collectively to large network input.

#### 1.2. Excitatory-Inhibitory networks with deterministic and noise driven dynamics

**Fig 3:**
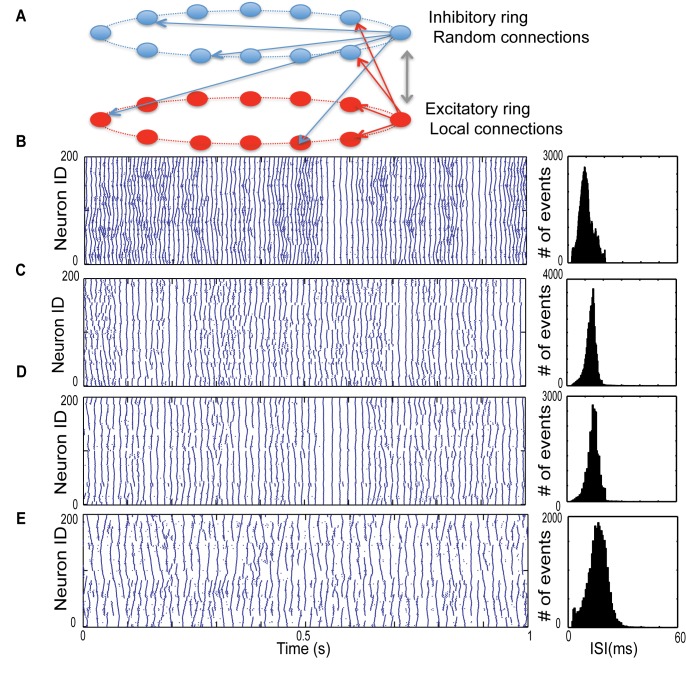
Interaction of excitatory and inhibitory networks for varying inhibitory connectivity in networks with deterministic dynamics. (A) Topology of interacting network of excitatory and inhibitory neurons. Here P_e_ = 0.15 and inhibitory connectivity changes from local (P_I_ = 0) to random (P_I_ = 1). (B) Excitatory only neurons with P_e_ = 0.15 when there is no inhibitory feedback. (C) P_i_ = 0, (D) P_i_ = 0.2, the local propagating waves in the asynchronous regime are destroyed. (E) Random inhibitory connections (P_i_ = 1), the firing frequency reduces significantly, while the propagating waves are longer and the synchronous bursting is suppressed. Spike timings used for analysis in this figure is provided as the supplemental data in S9–S12 Dataset.

Next, we investigated how the various topologies of inhibitory connectivity affect the network’s spatio-temporal patterning. In order to do so, we created two corresponding rings of excitatory and inhibitory cells. The inhibitory neurons send same number of connections as excitatory neurons to other inhibitory and excitatory neurons, but their synapses are weaker than those originating from excitatory neurons. Inhibitory neurons are connected using the same framework as excitatory cells—initially these neurons are connected locally and then rewire part of those local connections based on the inhibitory rewiring parameter (P_i_), Fig 3A. To look at the effect of inhibitory network’s connectivity pattern on the excitatory dynamics, we kept the excitatory rewiring parameter (P_e_) fixed and varied the inhibitory rewiring parameter (P_i_ = 0–1). Fig 3 presents results for the case when P_e_ = 0.15. In case of local inhibition (Fig 3C) we observed a strong suppression of propagating chains of activity in excitatory network in comparison with the excitatory only network dynamics (Fig 3B). This suppression is evident during asynchronous activity regimes. The shape of synchronous burst does not change significantly.

The increase of the inhibitory rewiring parameter (P_i_) causes complex changes to the spatio-temporal firing pattern of the excitatory cells (Fig 3C–3E). The firing chains within the asynchronous dynamics increase in length, however at the same time the synchronous bursts become suppressed for high P_i_ values (see Fig 3E). Furthermore, the overall frequency of the firing tends to decrease with P_i_. This is due to rapid spread and equalization of inhibition through out the network.

### 2. Identification and quantification of observed dynamical regimes

As we showed above, networks having different properties such as underlying structure, noise and different inhibitory connectivity pattern exhibit distinctive dynamics. In most regimes however we do observe periodic transitions from asynchronous (or less synchronized) to synchronous (or more synchronized) modes of activity. We set out to characterize these different patterns of activity and ultimately elucidate the predictive dynamical features of transitions between these dynamical regimes. In particular we want to investigate under what conditions (if any) these features can be identified sooner rather than later, and thus, reversing the question, can they tell us something about the underlying network properties.

Since the changes in network activity patterns are rapid, we cannot apply measures that are based on long temporal averages, as this would obscure the transition detection. Thus, to characterize the dynamics we developed a set of measures based on assessment of instantaneous changes in adjacent spike-timings of neurons. Based on the observations reported in previous section, the underlying idea of the proposed measures is to analyze, instead of changes in temporal distributions, instantaneous properties of spatial distributions of neuronal activity in given time windows. The major advantage of the developed metrics is that they are simple to compute based on the data that is readily available from recordings and thus can be applied directly to in vivo or clinical measurements. While the exact positions of the recorded cells are clearly unknown, one can ultimately divide the neural populations as coming from the same electrode (cells are nearby) and coming from other electrodes placed at various distances.

The specific question we want to answer is if, and if so, how much before the ultimate synchronous state can we detect changes in spatial network activity patterns. Also, we want to elucidate nature of this transition (e.g. is it a nucleation of locally synchronized groups of neurons)?

**Fig 4:**
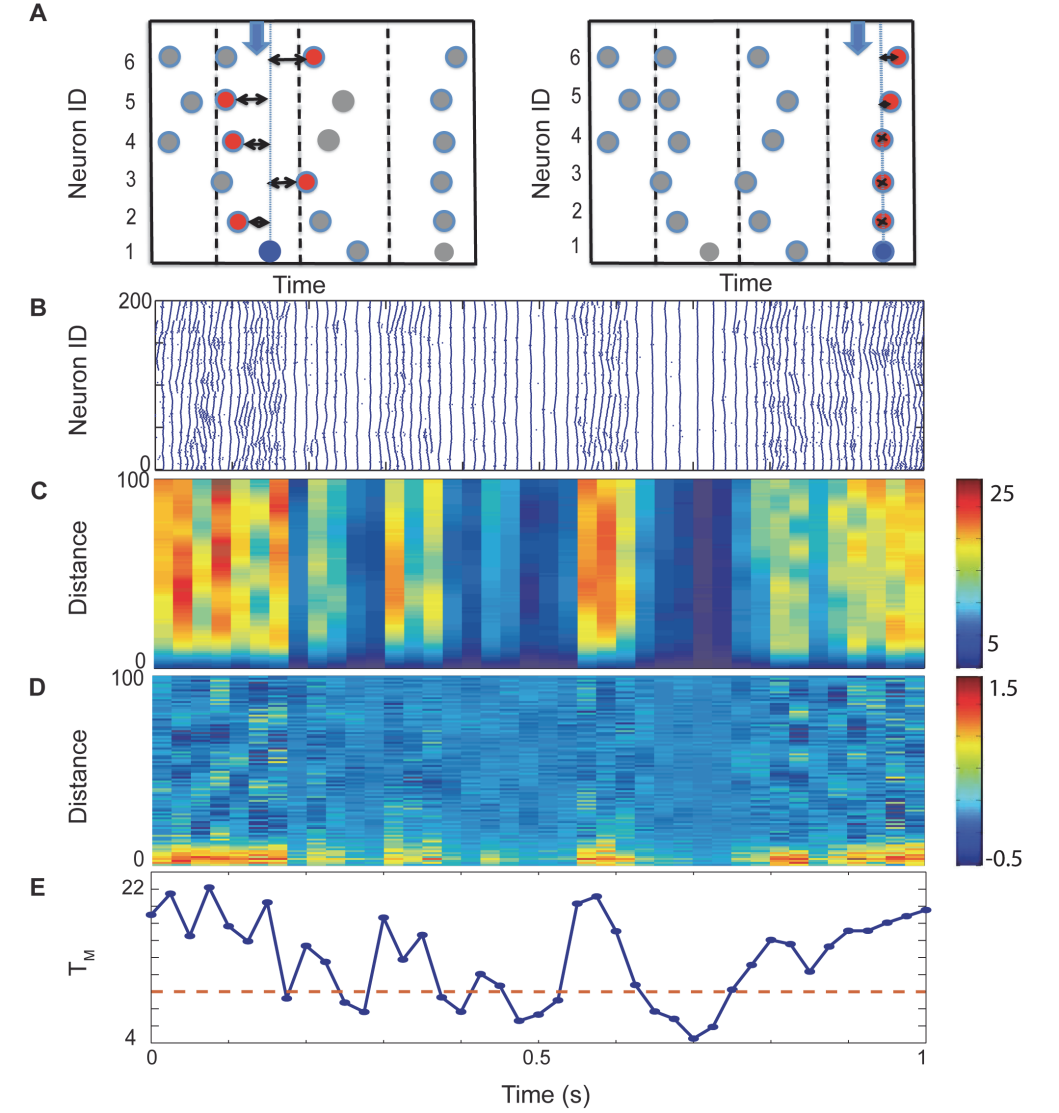
Characterization of the spatio-temporal dynamics of the network. (A) To characterize instantaneous spatial patterning in the network, we calculate the minimum time interval between each neuron’s spike in the time-window (blue filled circles) with all other neurons’ spikes and sort these timings based on their spatial distance. The left side of panel A shows the calculation for the a time window that has a asynchronous activity with relatively large and highly variable time intervals; the right side panel depicts calculation for the time window with a synchronous activity and minimal time differences between the spikes. (B) An example of raster plot obtained from noise driven excitatory only network P_E_ = 0.15, noise frequency f = 0.00005 (C) Color plot of consecutive T_D_ calculations; colors indicate the closest timing between spikes of neurons in the given window with all other neurons in the network. (D) Spatial derivative of T_D_ (dT_D_) in each time window. (E) Mean of T_D_ for consecutive time windows (to which we refer as T_M_). The dotted line is a cutoff, which we will use to identify the initiation of the bursting dynamics.

Here we divided the spiking data is divided into equal size time-windows with their duration matching the mean ISI observed in the network. Next, we calculate the time difference between closest (temporarily) spikes of every cell that fired within given window and every other cell in the network. These spike timings are then sorted based on the actual spatial distance of neurons (Fig 4A)—below we will refer to this vector as T_D_. We then aim to statistically characterize the properties of this vector as a function of network state, and more importantly near the impending transition into bursting.

Fig 4 shows an example of the raster plot (Fig 4B) and computed T_D_ vector for the consecutive time windows (Fig 4C). The color scale denotes the time difference between the spikes (note that scales are significantly different for different network structures). Fig 4D depicts the spatial derivative of T_D_ (dT_D_), while Fig 4E is the mean of T_D_ for a given time window (T_M_). We will use the T_M_ to identify the onset and offset of the bursting regime. We do this by setting a threshold value of T_M_ below which we considered that the network dynamics is largely synchronous. While this is to some extend arbitrary the results presented below are (within a range) largely independent of the exact value of the threshold chosen. The dotted line on Fig 4E denotes the threshold of transition between the two (asynchronous and bursting) regimes. One can easily observe, that the values of T_D_ are highly dependent on the distance between the cells. The universal property for all network structures (except when P_e_ = 1, see below) is the rapid loss of this distance dependence during the transition. We aim to statistically analyze and characterize properties of these transitions.

**Fig 5:**
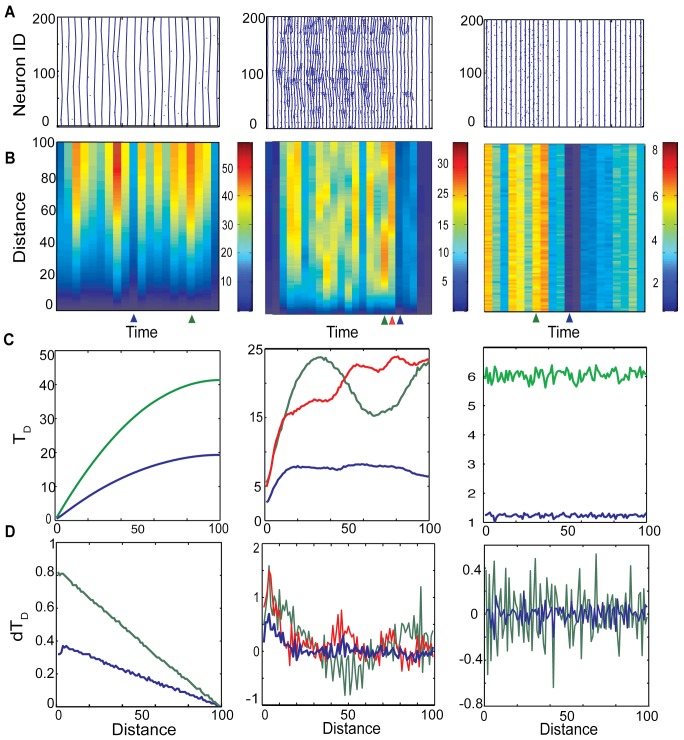
Raster plots and corresponding T_D_ and dT_D_ for selected time windows in an excitatory network. Where the left, middle and right column are associated with P_e_ = 0, P_e_ = 0.15, P_e_ = 1 respectively. (A) Raster plots; (B) Spatio-temporal changes of T_D_; (C) Examples of T_D_ evolution with distance for selected time windows (marked of B); (D) examples of derivative of T_D_ at the same timepoints.

The developed metric is quite sensitive to the changes in the network dynamics across various network structures and detects even small variations in the overall observed pattern of activity. An example of such is presented in Fig 5. This figure depicts changes in relative neurons’ firing pattern as a function of their relative distance reported by the T_D_, for various connectivity structures of excitatory network (P_e_ = 0; 0.15; 1.0). While the network spatio-temporal patterns are significantly different in the three cases, the metric picks up the bursting regime without difficulty. Moreover the internal structure of the T_D_ vector can shed the light on the intra-burst dynamics of the network. The spatial extend of the changes in T_D_ provides information about the correlation lengths between neuronal activities generated by propagating waves in the network. Thus when all connections are local and the average timing difference between spikes of neurons grows with their actual distance consistent with the long traveling chains of neuronal activities (Fig 5A). On the other hand, when the network has small-world connectivity pattern (P_e_ = 0.15), we observe much more complex correlation structure with significantly decreased correlation length. The distribution of the local extrema in the T_D_ again corresponds to the shorter chain lengths of activity in the raster plot (Fig 5B). Finally, when all the connections are random (P_e_ = 1.0), one can still observe changes in timing differences allowing for differentiation of dynamics between less and more synchronous network states. However there is no internal correlation within the given T_D_. In Fig 5C we pick few time-windows and show how these relative timings change as a function of actual distance for different structures: local, small-world and random. In case of local connections these timings increase monotonically with increasing spatial distance while in small-world structure there are local maxima and minima corresponding to the size of broken traveling chains. Finally in case of soley random connections there is no clear relationship between spike timings and spatial distance. In Fig 5D we showed changes in spatial derivatives for the same time windows.

#### 2.1. Characterization of dynamical network regimes using the developed metrics

**Fig 6:**
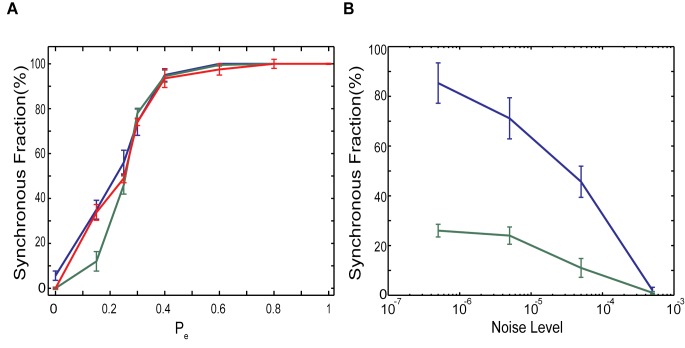
Characterizing the effect of noise level and connectivity structure on the dynamics of excitatory network. (A) Fraction of time network adopts synchronous dynamics as a function of rewiring parameter for noise driven identical (I^e^_ext_ = 1.05 for all neurons f_N_ = 0.00005, blue line), non-identical (I^e^_ext_ = 0.95–1.15, f_N_ = 0.00005, green line) and deterministic dynamics for network of non-identical cells (I^e^_ext_ = 0.95–1.15, red line). (B) The effect of the increasing noise level on the dynamics for the excitatory-only network with P_e_ = 0.15 for noise driven identical (I^e^_ext_ = 1.05 for all neurons, blue line) and non-identical (I^e^_ext_ = 0.95–1.15, green line).

First we set out to investigate the duration of the two (asynchronous and bursting) network regimes. We use the evolution of T_M_ to detect network durations in respective regimes. The threshold defining the onset of the bursting regime is set arbitrarily, however its specific value did not influence significantly the obtained results. We studied duration of the bursting regime for both excitatory only and excitatory-inhibitory networks as a function of topologies of both networks and also as a function of noise level. Fig 6 shows the fraction of time spent in the bursting regime for excitatory only networks as a function of these parameters. Fig 6A reports this fraction as a function of network connectivity (P_e_) for three types of networks. The first type are the networks composed of identical neurons (same driving excitatory current I_e_ = 1.05; see methods) having transitions between synchronous and asynchronous dynamics driven by the noise (f_N_ = 0.00005). The second network type is not driven by noise, but at the same time its elements are not identical in terms of their driving current and thus their intrinsic firing frequency (I_e_ = 0.95–1.15; note that the mean I_e_ = 1.05). Eventually the third one is driven by noise and its neurons are non-identical in terms of the driving current (f_N_ = 0.00005, I_e_ = 0.95–1.15; mean I_e_ = 1.05, green line). As observed earlier (Fig 1) the fraction of time spent in synchronous regime increases significantly with increasing P_e_. At the same time, for small-world regime, heterogeneity of neurons along with noise (Fig 6A, green line) considerably lowers the fraction of time spent in synchronous dynamics. However, there is no significant difference between noise driven transitions and those cause internally by cell heterogeneity. For higher P_e_ values (P_e_>0.3), the neuronal heterogeneity nor/and noise does not change the duration of bursting dynamics significantly.

On the other hand the effect of noise on the dynamics for the excitatory network is illustrated in Fig 6B. Here we vary the noise level (i.e. the probability of occurrence of random spikes) for the two networks: those having identical driving current applied to all cells (blue line) and those having driving current randomly chosen from a distribution I_e_ = 0.95–1.15 (green line). The fraction of time spent in synchronous dynamics is suppressed with the increased levels of noise, but also depends strongly on cellular heterogeneity.

**Fig 7:**
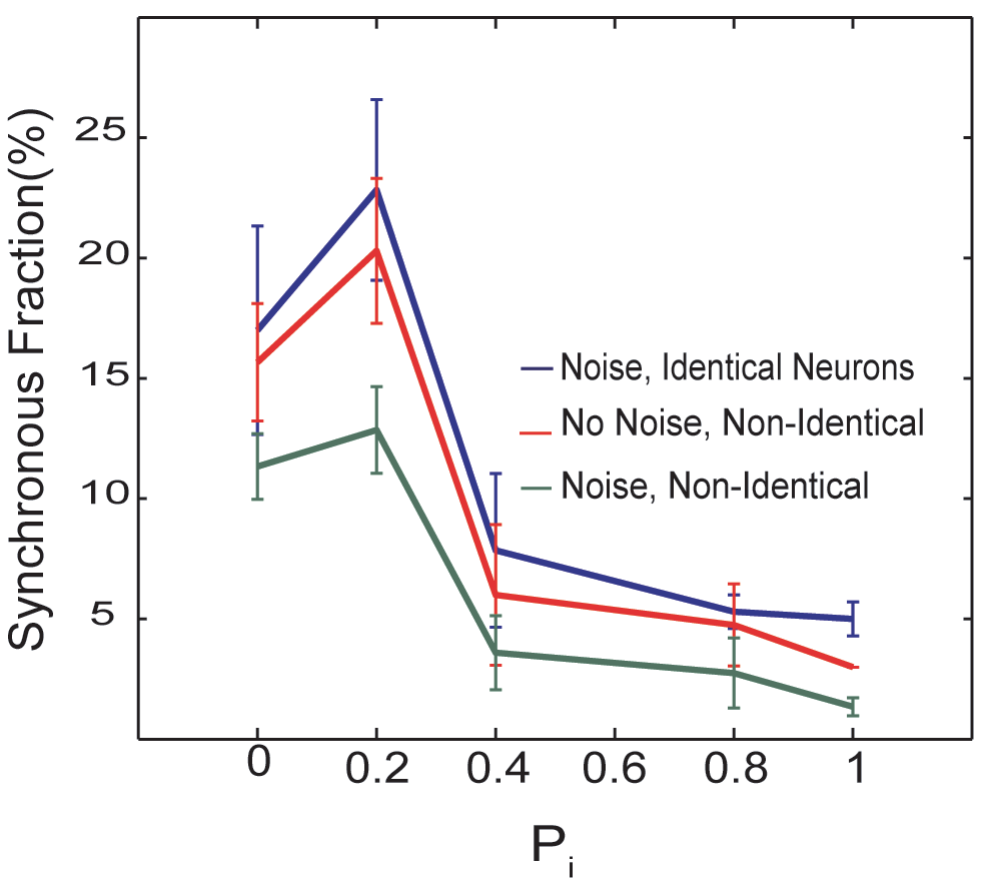
Fraction of time that the network spent in the synchronous regime. The synchronous fraction of dynamics as a function of inhibitory connectivity when excitatory connectivity is in small-world regime (P_e_ = 0.15), for: 1) noise driven identical neurons (f_N_ = 0.00005, I_e_ = 1.05,I_i_ = 0.95; blue line), 2) non-identical neurons (f_N_ = 0.00005, I_e_ = 0.95–1.15, I_i_ = 0.9–1.0; green line), and 3) deterministic dynamics of non-identical neurons (no noise, I_e_ = 0.95–1.15, I_i_ = 0.9–1.0; red line).

We analyzed in similar fashion the effect of the inhibitory topology on the spatio-temporal dynamics of the excitatory network (Fig 7). As before we investigated the dynamics of three types of networks—the noise driven dynamics of networks composed of either identical neurons, non-identical neurons and fully deterministic dynamics of networks composed of non-identical cells (both excitatory and inhibitory). The mean driving current of the inhibitory cells was set to be I_i_ = 0.95—effectively below spontaneous firing threshold. Thus their firing was driven only by the excitatory network and/or noise. The connectivity of the excitatory networks was kept constant at P_e_ = 0.15 and we varied the inhibitory connectivity (P_i_ = 0.0–1.0). Interestingly the small-world regime of inhibitory connectivity corresponds to the largest fraction of time spent in synchronous dynamics (Fig 7). As expected overall fraction of time that network spent in synchronous dynamics is lower in the presence of both noise and heterogeneity (green line). It is interesting to note that network spends most time in synchronous bursting regime when P_I_ = 0.15 (small world topology) and it significantly decreases for random inhibitory network structure. This could indicate that changes in overall inhibitory network structure for example due to axonal sprouting could lead to network more prone to bursting.

#### 2.2. Network transitions from asynchronous to bursting regime

The ultimate goal of this study is to characterize network transitions and their predictability from asynchronous activity into the bursting regime. Here we limit the meaning of predictability to identification of first signs of transition to bursting dynamics before the transition itself takes place. Thus, we setout to identify the predictive dynamical features of the transitions as well as their first occurrence relative to the closest transition time, through further analyzing of the T_D_ vector near the transition points. Specifically we utilize measures such as T_M_ (mean value of all T_D_ values), variance from the mean of T_D_ values and variance of dT_D_ (the spatial derivative of T_D_, see Figs 4 and 5) to detect precursors of the transition preceding the bursting onset. As we will show below, we observed systematic changes in these measures prior to the onset of transitions into the bursting. These changes can be interpreted as the early features of incoming transition (or beginnings of the transition itself) and based on that we can obtain lead-time estimate to the instance when fully synchronous state takes hold (see methods). We characterize this transition predictability as a function of topologies of both excitatory and inhibitory networks, heterogeneity of cells and noise levels.

We want to use measures characterizing properties of T_D_ to detect precursors of the transitions into the bursting dynamics and calculate the lead-time T_L_ (or predictability) to the transition, as a time period before the transitions, during which we can detect significant changes in dynamics, as reported by the developed metrics. First, we measure the values of the above defined metrics in the time windows immediately preceding the onset of the bursts (as defined by the T_M_). We then calculate the ratios of these values obtained in the consecutive time windows. Thus, we calculate R_N_ = M_N+1_/M_N,_ where R_N_ denotes the ratio of the (generalized) measure ‘M’ calculated ‘N’ time-windows before the burst onset (N = 0, 1, 2, 3, 4, 5). We then average the ratios over all the realizations of transitions for given network type. If the R_N_ is significantly different from unity we assume that spatial patterning within this time window is persistently and significantly different from that in the prior window. At the same time, the lead-time is defined as the number of time windows prior bursting onset within which the spatio-temporal network pattern undergoes significant change with respect to the one observed in a window before. We defined “predictability” or Lead-time as a number of windows prior to the onset of bursting when the ratio is significantly different from one.

**Fig 8:**
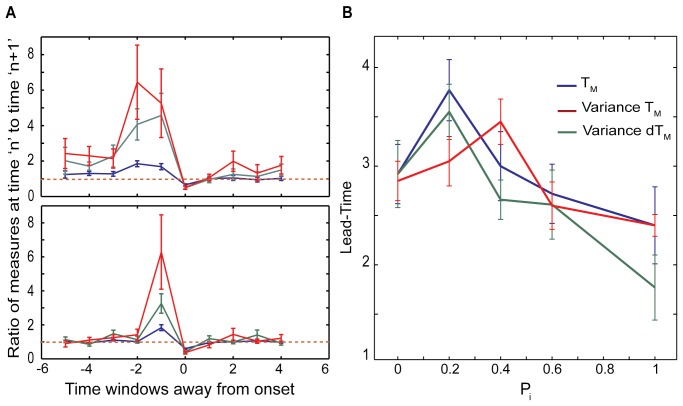
The effect of inhibitory connectivity on the lead-time, T_L_. (A-B) the ratio of measures (T_M_: blue line, Variance Of T_M_: red line, Variance Of dT_M_: green line) before and after the onset of the transition into the bursting is shown for P_i_ = 0.2 and 1, respectively; P_e_ = 0.15, f_N_ = 0.00005. Based on these ratios, T_L_ is calculated as a function of the inhibitory connectivity pattern (C). T_L_ peaks for P_i_ = 0.2 and then decreases for more random inhibitory topologies.

**Fig 9:**
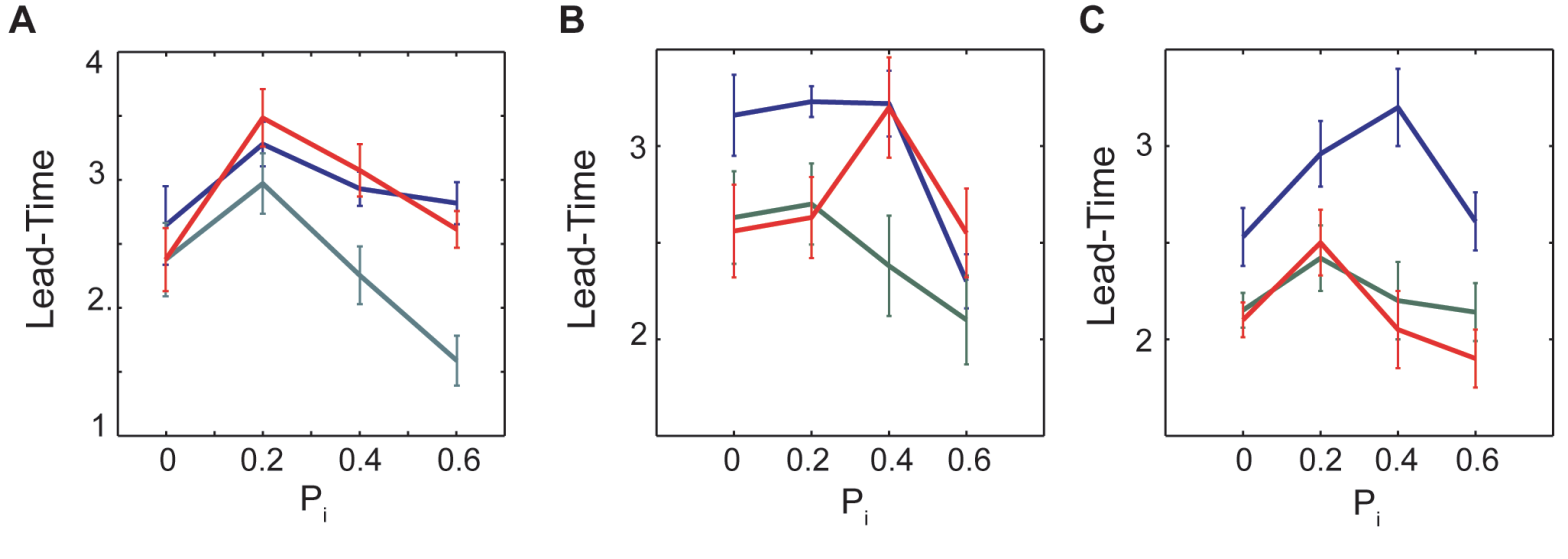
T_L_ varies as a function of inhibitory connectivity (P_i_) for excitatory networks with different values of P_e._. (A) P_e_ = 0.0, (B) P_e_ = 0.15 and (C) **P**_e_ **= 1**.0 where blue, red and green lines are standing for obtained T_L_ based on the T_M_, variance of T_M_ and variance of dT_M_ measures respectively.

**Fig 10:**
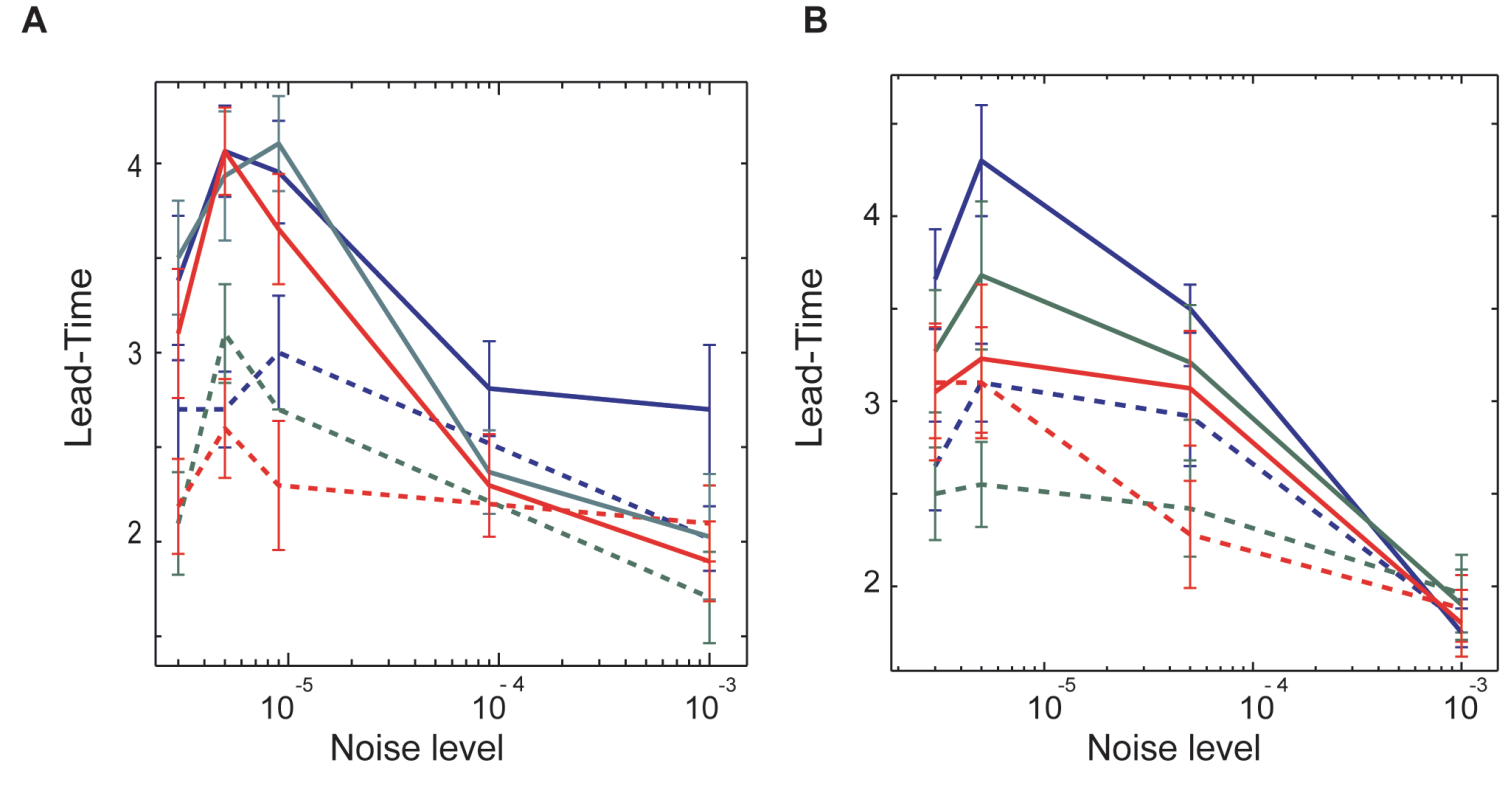
The effect of noise on the lead-time of the transitions for both excitatory and interacting excitatory-inhibitory networks. (A) Excitatory networks (P_e_ = 0.15); (B) excitatory and inhibitory networks (P_e_ = 0.15, P_i_ = 0.0). Solid lines denote simulations in which all neurons receive identical external current (I_e_ = 1.05, I_i_ = 0.95), while dashed-lines are representing simulations with distribution of external currents (I_e_ = 0.95–1.15, I_i_ = 0.9–1.0).

Fig 8 depicts estimation of the lead-time as a function of inhibitory network connectivity. The vertical dashed line (Fig 8A and 8B) denotes the transition point into bursting dynamics. We report the ratios of three derivative measures of the T_D_ vector (T_M_, spatial variance of T_D_ and its spatial derivative, dT_D_ vector, for given time-window) calculated in the times windows N+1 and N before and after the transition (N = 0, 1, 2, 3, 4, 5). Fig 8C shows lead-time (T_L_). All three measures used show significant changes before the transition to bursting. The changes in variance of both T_D_ and of dT_D_ show the largest changes before the transition point. However in terms of estimated lead-time T_M_ performs somewhat better (see also Figs 9 and 10).

The excitatory network topology is fixed (P_e_ = 0.15) and neurons are identical (I_e_ = 1.05) driven by noise (f_N_ = 0.00005). The connectivity pattern of the inhibitory network is being changed from local (P_i_ = 0) to random (P_i_ = 1). The examples of the ratios of the three metrics at the consecutive time-windows are depicted for P_i_ = 0.2 (Fig 8A) and P_i_ = 1 (Fig 8B). We observe that depending on the inhibitory topology all three measures report different predictability intervals—with longer ones being reported for P_i_ = 0.2. Fig 8C reports the lead-time as a function of the inhibitory network connectivity. As mentioned before the size of time windows depends on spiking frequency. If we assume that the mean spiking rate is around 10- 20Hz the lead-time can be estimated to be up to 200-400ms (4 time-windows). While not a lot, this maybe enough to provide electrical stimulation to disrupt pathological pattern.

To better understand the interaction of the excitatory and inhibitory topologies on the lead-time (T_L_), we explored the effect of inhibition on networks with three excitatory connectivity patterns P_e_ = 0.0, 0.15 and 0.4 having deterministic dynamics (I^e^_exc =_ 0.95–1.15, I^i^_exc_ = 0.9–1, Fig 9). Here the general trend is less clear, but there is a moderate decrease of the lead-time for more random inhibitory topologies, with shift of the predictability peak towards values where P_e_≅P_i_, as reported by T_M_.

We next used the measures described above to characterize the effect of noise and variability of neuronal firing frequency on the transition lead-time for both, the excitatory (Fig 10A) and interacting excitatory-inhibitory (Fig 10B) networks. The solid-lines are for the case when neurons are receiving the same external current, while dashed lines are for the case when the neurons have significantly different intrinsic firing frequencies as their driving current varies between I_e_ = 0.95–1.15 for excitatory cells and I_i_ = 0.9–1.0 for inhibitory cells. Obtained results suggest that predictability is much higher for the networks in which neurons have similar intrinsic firing frequencies, however as expected the lead-time decreases with the increasing noise level.

## Methods

### Spiking neuron model

The Leaky integrate-and-fire (LIF) model was used to simulate network of excitatory and interacting excitatory-inhibitory neurons. The evolution of the voltage across the membrane of neuron ‘j’ is defined as follows:

$$C\frac{dV^{j}}{dt}=-\alpha^{j}(V^{j}-E)+I_{ext}+I_{syn}^{j}$$ (1)

Where V^j^ and C are the voltage and capacity across j^th^ neuron’s membrane, respectively. The constant α is the leak conductance of the cell that is minimally different for each neuron and chosen from Gaussian distribution (μ = 1, SD = 0. 05). Here I_ext_ is an externally applied current to each cell. Depending on the network model studied (i.e. whether the transitions are due to the noise or to the significant firing frequency mismatch) it can be identical for all of the neurons—external current that excitatory neuron receives: I^e^_exc_ = 1.05 (the steady state is for most cells just above threshold), external current that inhibitory neuron receives: I^i^_exc_ = 0.95 (just below the threshold for most cells), or it can be taken from a uniform distribution (I^e^_exc_ = 1.05, SD = 0. 1 and I^i^_exc_ = 0.95, SD = 0. 05). After the electrical potential across the cell membrane achieves the threshold set to V_T_ = 1, the cell fires an action potential and its membrane potential is reset to V_reset_. We set resting-potential ‘E’ and reset-potential ‘V_reset_‘ equal to zero. Immediately after neuron spikes, the cell enters the refractory period (T_ref_ = 1.5ms).

This synaptic input from presynaptic cell into the postsynaptic cell can be positive or negative depending on its excitatory or inhibitory character of the presynaptic cell and is defined as follows:

$$I_{syn}^{j}=\omega \sum_{i}^{}A_{ij}\left(H(t)-H(t-\tau )\right)$$ (2)

Where i and j are presynaptic and postsynaptic neurons, respectively. The ω is the efficacy of connection between presynaptic and postsynaptic neurons; A is the adjacency matrix, H(t)—a Heaviside function, and τ = 1 ms represents the spike duration. We used Euler method with step size h≈0.01ms (estimated from time duration of the spike) to integrate LIF equation for the network.

For networks dynamics incorporating stochastic component, we defined noise as a lighting bolt arriving randomly at each cell with predefined probability. Its arrival at a given site caused the cell to fire instantaneously independent of the membrane voltage, unless the cell was currently spiking or in its refractory time.

### Networks Structures

The excitatory only network is composed of 200 excitatory neurons forming a 1-D ring structure. The small-world framework was used to vary continuously the network connectivity depending on the rewiring probability [34]. This rewiring breaks a local connection that comes from presynaptic neuron and forms a new random connection to any other postsynaptic neuron that didn’t have connection before. Thus the network connectivity can vary from local connectivity (P_e_ = 0), to the random connectivity (P_e_ = 1). Every neuron establishes 8 connections to its neighbors (i.e. R = 4).

For interacting excitatory and inhibitory systems we added a corresponding network of 200 inhibitory cells. Thus here the network connectivity pattern is defined by two parameters (P_e_ and P_i_). Every excitatory cell makes 8 connections to other excitatory and inhibitory cells, every inhibitory cells makes also 8 connections to both other inhibitory and excitatory neurons. The synaptic weight for connections originating from excitatory cells is ω_e_ = 2.2, while that of inhibitory neurons ω_i_ = 0.8.

### Analysis

In order to identify type of the networks’ dynamics (asynchronous versus synchronous) and characterize their transitions, we created a measure based on the relative timing of each neuron’s with respect to other neurons in the system (T_D_). We divided time of simulation into number of equal length time-windows. The length of the time-window was set to be the average spike frequency in the network. At each time window, the minimum time difference between every neuron’s spike within that window and all other cells is computed (regardless whether the other cells spike within that time-window). If there is more than one spike per neuron in a time window we choose the earliest spike’s time for the given neuron. This calculation is repeated for all the consecutive time windows. These times are then sorted based on the physical distance between neurons and the histogram of the mean times at every distance is resulting in distinct spatial vector T_D_ generated for every time window. The example of T_D_ can is illustrated using a color plot on Fig 4, where vertical axis represents the distance between two neurons, horizontal axis is showing the time of simulation. The color scale indicates the T_D_ values.

We define T_M_ as (spatial) average of T_D_ and use its value to detect temporal location of the transitions into and out of the bursting regime. To characterize the properties of the T_D_ vector near the transition point we calculate its spatial derivative dT_D_. Finally we calculate dT_M_, which is the average value of dT_D_ and also variance of T_D_ and dT_D_.

### Onset and slope of the transition

We want to use measures characterizing properties of T_D_ to detect precursors of the transitions into the bursting dynamics. We calculate the values of the above defined measures in the time windows immediately preceding the onset of the bursts (as defined by the T_M_). We then calculate the ratios of the measures progressing forward in time. Therefore we calculate R_N_ = M_N+1_/M_N_), the ratio of the (generalized) measure ‘M’ calculated ‘N’ time-windows before the burst onset (N = 0, 1, 2, 3, 4, 5). If the R_N_ is significantly different from unity we assume that spatial patterning within this time window is persistently and significantly different from that in the prior window. The lead-time is defined as the number of time windows prior bursting onset within which the spatio-temporal network pattern undergoes significant change with respect to the one observed in a window before. This lead-time is then averaged over many realizations of bursting transitions.

## Discussion

In this study we investigated predictability of network transitions into bursting regime as a function of network structure, cell variability and noise. Initially, we characterized the dynamics for different parameter sets and then we used the developed measures to predict transitions to synchronous activity using spike timings. The networks, as predicted exhibit different types of dynamics, ranging from propagating waves of activity, through coexistence of two phases with short waves of activity and bursting, and finally synchronous dynamics. Addition of inhibition to network shortens the propagating waves, with the transition to bursting suppressed for random inhibitory topologies.

Over the last few decades amount of research is associated with finding robust measures that can detect synchrony [50–53]. In general these measures require relatively long time series, making them not applicable to measure relatively rapid transitions in network patterning, such as the onset of seizure. The metrics that we propose here aims to detect instantaneous changes in spatial statistics of spiking coincidence.

The introduced measures centered on analysis of relative spike timings of all firing cells within a given time window. The metrics characterized instantaneous spatial correlations between the cells as a function of their physical distance. The systematic changes in the introduced measures in the time windows preceding the bursting onset, were able to predict transition into bursting within few time windows of its onset. It is important to note however that the approach taken does not allow estimating the false positives (i.e. when observed change does not lead to bursting transition), resolving these changes from the ones leading to bursting onset is a subject of ongoing research. The performance of the metrics depended on network topology, noise level and distribution of cellular firing rates. The constructed metrics provide an alternate approach toward gaining an insight on transitions between asynchronous and bursting dynamics. Their advantages are that they can be computed rapidly and thus applied online in clinical use.

## Supporting Information

S1 Dataset (Fig 1A) Spike-timings. Excitatory only network, P_e_ = 0, neurons receive no noise, and their external current is taken from a uniform distribution of I^e^ext = 0.95–1.15.
(XLS)

S2 Dataset (Fig 1B) Spike-timings. Excitatory only network, P_e_ = 0.15, neurons receive no noise, and their external current is taken from a uniform distribution of I^e^ext = 0.95–1.15.
(XLS)

S3 Dataset (Fig 1C) Spike-timings. Excitatory only network, P_e_ = 0.4 neurons receive no noise, and their external current is taken from a uniform distribution of I^e^ext = 0.95–1.15.
(XLS)

S4 Dataset (Fig 1D) Spike-timings. Excitatory only network, P_e_ = 1, neurons receive no noise, and their external current is taken from a uniform distribution of I^e^ext = 0.95–1.15.
(XLS)

S5 Dataset (Fig 1E) Spike-timings. Excitatory only network, P_e_ = 0, with noise frequency of fN = 0.00005 and all neurons receive identical external current I^e^ext = 1.05.
(XLS)

S6 Dataset (Fig 1F) Spike-timings. Excitatory only network, P_e_ = 0.15 with noise frequency of fN = 0.00005 and all neurons receive identical external current I^e^ext = 1.05.
(XLS)

S7 Dataset (Fig 1G) Spike-timings. Excitatory only network, P_e_ = 0.4, with noise frequency of fN = 0.00005 and all neurons receive identical external current I^e^ext = 1.05.
(XLS)

S8 Dataset (Fig 1H) Spike-timings. Excitatory only network, P_e_ = 1, with noise frequency of fN = 0.00005 and all neurons receive identical external current I^e^ext = 1.05.
(XLS)

S9 Dataset (Fig 3A) Spike-timings. Excitatory only network, P_e_ = 0.15, neurons receive no noise, and their external current is taken from a uniform distribution of I^e^ext = 0.95–1.15.
(XLS)

S10 Dataset (Fig 3B) Spike-timings. Excitatory neurons in the excitatory-inhibitory network, where P_e_ = 0.15 and P_i_ = 0, neurons receive no noise, and their external current is taken from a uniform distribution of I^i^ext = 0.9–1, I^e^ext = 0.95–1.15.
(XLS)

S11 Dataset (Fig 3C) Spike-timings. Excitatory neurons in the excitatory-inhibitory network, where P_e_ = 0.15 and P_i_ = 0.2, neurons receive no noise, and their external current is taken from a uniform distribution of I^i^ext = 0.9–1, I^e^ext = 0.95–1.15.
(XLS)

S12 Dataset (Fig 3D) Spike-timings. Excitatory neurons in the excitatory-inhibitory network, where P_e_ = 0.15 and P_i_ = 1, neurons receive no noise, and their external current is taken from a uniform distribution of I^i^ext = 0.9–1, I^e^ext = 0.95–1.15.
(XLS)
